# Swimming performances in long distance open-water events with and without wetsuit

**DOI:** 10.1186/2052-1847-6-20

**Published:** 2014-05-21

**Authors:** Sebastian Ulsamer, Christoph Alexander Rüst, Thomas Rosemann, Romuald Lepers, Beat Knechtle

**Affiliations:** 1 Institute of General Practice and for Health Services Research, University of Zurich, Zurich, Switzerland; 2 INSERM U1093, Faculty of Sport Sciences, University of Burgundy, Dijon, France; 3 Facharzt FMH für Allgemeinmedizin, Vadianstrasse 26, Gesundheitszentrum St. Gallen, St. Gallen, Switzerland

**Keywords:** Ultra-endurance, Swimming, Ironman, Neoprene suit, Swim performance

## Abstract

**Background:**

Existing literature showed improved swimming performances for swimmers wearing wetsuits competing under standardized conditions in races held in pools on short to middle distances. Data about the influence of wetsuits on swimming performances in long and ultra-long open-water swimming races are missing. It is unknown whether the benefit of wearing wetsuits is comparable in men and women. The aim of this study was to investigate the influence of wearing a wetsuit on open-water swimming performances at the 26.4 km ‘Marathon Swim in Lake Zurich’ in Lake Zurich, Switzerland, and the 3.8 km Lake Ontario Swim Team-Race (LOST-Race) in Lake Ontario, Canada.

**Methods:**

Race times of the fastest female and male swimmers competing with and without wetsuit were compared using multi-level regression analyses and analysis of variance.

**Results:**

In the ‘Marathon Swim’ in Lake Zurich, wearing a wetsuit had no effect on race time regarding the gender where athletes wearing a wetsuit were not faster than athletes without wetsuit. However, the ten fastest men wearing a wetsuit (410.6 ± 26.7 min) were faster (32.7%, p < 0.01) than the ten fastest women without wetsuit (544.9 ± 81.3 min). In the ‘LOST-Race’, the top ten men wearing a wetsuit (51.7 ± 2.5 min) were faster (13.2%, p < 0.01) than the top ten women wearing a wetsuit (58.5 ± 3.2 min). Additionally, the top ten men without wetsuit (52.1 ± 2.4 min) were faster (19.6%, p < 0.01) than the top ten women without wetsuit (62.3 ± 2.5 min). The top ten women wearing a wetsuit (58.5 ± 3.2 min) were faster (6.5%, p < 0.01) than top ten women without a wetsuit (62.3 ± 25 min).

**Conclusions:**

These results suggest that wearing a wetsuit had a positive influence on swimming speed for both women and men but the benefit of the use of wetsuits seemed to depend on additional factors (*i.e.* race distance). Women seemed to benefit more from wearing wetsuits than men in longer open-water ultra-distance swimming races.

## Background

Over the past decade the popularity of participation in ultra-endurance events such as ultra-marathon running [1-4], ultra-cycling [5], ultra-triathlons [6,7] and ultra-swimming [8-10] has considerably increased. For ultra-endurance swimmers, several events such as the ‘Manhattan Island Swim’ in New York [11], the ‘Rottnest Channel Swim’ in Australia [12], the ‘Marathon Swim’ in Lake Zurich in Switzerland [13] or the ‚English Channel Swim’ between Dover and Calais [14] are held. Recent studies showed that women reduced the sex difference in open-water ultra-distance swimming [15,16] and were even able to outperform men [17].

Long-distance swimming splits are also included in other ultra-endurance events such as long-distance triathlons (*i.e.* Ironman triathlon) consisting of 3.8 km swimming, 180 km cycling and 42 km running [7]. The same 3.8 km swimming distance as held in Ironman competitions is covered by swimmers competing in the ‘LOST-Race’ (*i.e.* Lake Ontario Swim Team-Race), an open-water swimming race held in the Lake Ontario, Canada. The ‘LOST-Race’ is organized since 2008 by the Lake Ontario Swim Team [18]. When the race started in 2008, only eight swimmers participated but the number of swimmers increased continuously up to 94 starters in 2012 [19].

Generally, open-water ultra-swimmers are prohibited to wear wetsuits in open-water swimming events such as the‚ ‘English Channel Swim’, where the athletes have to cover a total distance of ~32 km [9]. In contrast to open-water ultra-distance swimming, participants at the ‘Marathon Swim’ in Lake Zurich, Switzerland, the longest open-water ultra-swim in Europe held in a lake covering a distance of ~26.4 km [13], are allowed to wear wetsuits when competing in the wetsuit category. These participants are separately ranked in a wetsuit category. Also the participants in the ‘LOST-Race’ are free to decide if they want to start in the wetsuit or in the non-wetsuit category [18].

In official Ironman competitions the permission of the use of wetsuits depends on the water temperature. Wetsuits are permitted when the water temperature is up to 24.5°C or colder but will be prohibited in water temperatures higher than 28.8°C. Athletes who choose wearing a wetsuit in water temperatures between 24.5°C and 28.8°C are allowed to participate but are not eligible for age-group awards or for qualifying slots for the Ironman World Championship [20].

When triathletes began wearing wetsuits during the swimming part of a triathlon race, anecdotal evidence as well as the empirical study of Parsons and Day [21] indicated that the swimming performance could be improved by wearing a wetsuit. Studies have been conducted since then investigating the influence of wearing a wetsuit on open-water swimming performance [22,23]. It has been shown that wearing wetsuits enhanced swimming performance by directly increasing buoyancy and holding the body in a more horizontal position, thereby allowing more effort to be expended in propulsive movement [23]. The performance gain while wearing a wetsuit was associated with an increased propulsion efficiency related to both a gain in buoyancy [24] and a reduction in drag [25]. It seemed that swimming performance improved more in inefficient swimmers with low buoyancy and swimming at low speed [26]. Wearing a wetsuit reduced drag during front crawl swimming [27,28] leading to a decreased energy cost of submaximal swimming and an increased distance per stroke [27]. Differences do also exist between fastskin and standard swim wetsuits regarding drag and buoyance where full-length fastskin swimsuits created less total hydrodynamic resistance than normal swimsuits while providing no additional buoyancy benefits [29]. Also a torso swim suit and a standard racing suit lead to differences in drag and performance. A torso suit reduced the energy demand of swimming whereas a standard racing suit reduced body drag [30]. Also the length of the swimming performance seems of importance where performance was not enhanced in rather short swimming distances [31].

Actually, literature about the effects of a wetsuit on long-distance and ultra-long-distance swimming performance is very small [22]. In all studies investigating the effect of wetsuits on swimming performance, participants were swimming in pools under standardized conditions or covered only short to middle distances of 400 m and 1,500 m or short swim times of 5 min to 30 min [21,22]. Studies on the effect of wearing wetsuits on swimming performance in open-water ultra-distance swimming are missing so far. According to Knechtle *et al.*[32] investigating predictor variables in an open-water ultra-endurance swimming event covering ~26.4 km, none of the known anthropometric variables in swimmers such as body fat, body height and length of the extremities showed a relationship with race time. Regarding the fact that there are rather few variables with potential influence on ultra-swimming performance, a positive effect of the use of a wetsuit on race time in an ultra-endurance open-water swimming contest would be of great interest.

In open-water ultra-distance swimming, Eichenberger *et al.* reported faster race times for male swimmers competing in the ‘Marathon Swim’ in Lake Zurich [10] compared to race times in female finishers and mentioned physiological and anthropometric differences as potential reasons for the sex difference in performance. Only the study of Toussaint *et al.*[24] compared the effects of wearing a wetsuit on the performance of male and female swimmers where changes in swimming times were equal in male and female swimmers swimming with and without wetsuits. As this study has been conducted under standardized conditions while swimming in a pool, no study investigated possible differences of the benefit of wearing wetsuits between male and female swimmers in long and ultra-long open-water swimming.

Therefore, the aim of the present study was to compare the performance of female and male open-water swimmers competing with or without wearing a wetsuit in a long-distance (*i.e.* 3.8 km) and in an ultra-long-distance swimming (*i.e.* 26.4 km) event. We hypothesized that (*i*) swimmers wearing a wetsuit would finish the races in a shorter time than swimmers without wearing a wetsuit and (*ii*) women might be able to outperform men in the longer race distance (*i.e.* 26.4 km).

## Methods

All procedures used in the study were approved by the Institutional Review Board of Kanton St. Gallen, Switzerland with a waiver of the requirement for informed consent of the participants given the fact that the study involved the analysis of publicly available data.

### Data sampling

All data including race times and water temperatures was obtained from the official result lists published on the official homepages of the ‘Marathon Swim’ [33] in Lake Zurich, Switzerland, and the ‘LOST-Race’ [34] in Canada.

### The ‘Marathon Swim’ in Lake Zurich

The ‘Marathon Swim’ in Lake Zurich takes place annually in Lake of Zurich in Switzerland at the beginning of August. The first edition of the race was in 1987. The use of wetsuits has been allowed since 2002 in a separate category and swimmers can compete in a wetsuit and a non-wetsuit category. The swimmers start together at 7:00 a.m. from Rapperswil and have to cross the length of the lake and finish in Zurich. The race covers a distance of ~26.4 km where the cut-off time is 12 h. The use of high-tech swimsuits and additional floating devices was prohibited in the non-wetsuit category. Each swimmer has to be accompanied by a support boat. The swimmers have to organize their own nutrition and support crew during the race. All athletes who ever successfully finished the ‘Marathon Swim’ in Lake Zurich between 2002 and 2012 were analysed regarding the association between wearing a wetsuit, sex and swimming speed.

### The LOST-Race

The ‘LOST-Race’ is the abbreviation for the ‘Lake Ontario Swim Team’-Race. This race is held annually 2008 in the middle of August. The race starts at 8.00 a.m. from the foot of Maple Grove Drive for 3.8 km to the Lighthouse Pier in downtown Oakville. This distance is equal to the swim split in an Ironman triathlon. The swimmers can compete in a wetsuit and a non-wetsuit category. All athletes who successfully finished in the ‘LOST-Race’ between 2008 and 2012 were analysed regarding the association between wearing a wetsuit, sex and swimming speed.

### Data analysis

To examine the change in performance across years, race times of the annual top (*i.e.* swimmers with the fastest race time) and annual top three (*i.e.* swimmers with the three fastest race times) female and male swimmers competing with and without wetsuit were determined for both races. Due to the low number of female finishers in the ‘Marathon Swim’ in Lake Zurich women could only be included in the analysis of the annual top swimmers. When in the ‘LOST-Race’ less than three athletes were recorded per category in a certain year, the specific year was excluded from analysis for that specific group. The mean race times of both the annual top and the annual top three athletes were pooled and performance was compared within swimsuit groups (*i.e.* wearing a wetsuit or not) between sexes and between wetsuit groups within sexes. Additionally, the overall (*i.e.* all finishers between 2002 and 2012) top ever, top three ever and top ten ever women and men competing with and without wetsuit were determined and compared within wetsuit groups between sexes and within sexes between wetsuit groups. As a last step, the interaction between wetsuit and sex on performance was determined for the annual top swimmers as well as for the top, top three and top ten swimmers ever.

### Statistical analyses

Each set of data was tested for normal distribution and for homogeneity of variances prior to statistical analyses. Normal distribution was tested using a D’Agostino and Pearson omnibus normality test and homogeneity of variances was tested using a Levene’s test. Trends in participation were analysed using regression analysis with ‘straight line’ and ‘exponential growth equation’ model, whereas for each set of data (*e.g.* each age group) both models where compared using Akaike’s Information Criteria (AICc) to decide which model showed the highest probability of correctness. Single and multi-level regression analyses were used to investigate changes in performance of the finishers. A hierarchical regression model was used to avoid the impact of a cluster-effect on results in case one athlete finished more than once in the annual top or annual top three for the analysis of overall performance. Regression analyses of performance were corrected for age of athletes in ‘Marathon Swim’ in Lake Zurich to prevent a misinterpretation of an ‘age-effect’ as a ‘time-effect’. To find significant differences between two groups, a Student’s *t*-test was used with Welch’s correction in case of unequal variances. To determine the interaction of sex and wetsuit on swimming performance, a two-way ANOVA (sex × wetsuit) was performed. Statistical analyses were performed using IBM SPSS Statistics (Version 19, IBM SPSS, Chicago, IL, USA) and GraphPad Prism (Version 5, GraphPad Software, La Jolla, CA, USA). Significance was accepted at p < 0.05 (two-sided for *t*-tests). Data in the text are given as mean ± standard deviation (SD).

## Results

### The ‘Marathon Swim’ in Lake Zurich

**Figure 1:**
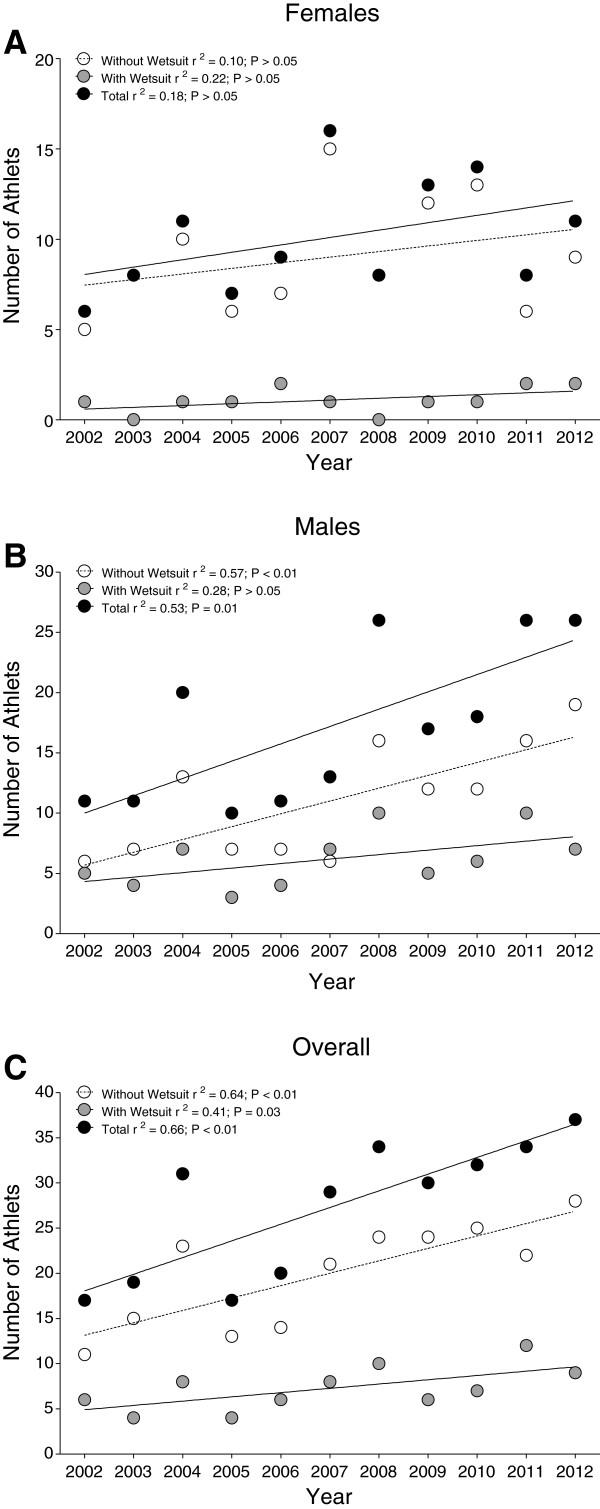
Annual number of finishers in the ‘Marathon Swim’ in Lake Zurich competing with and without wetsuit as well as total for women (Panel A), men (Panel B) and overall finishers (Panel C).

The participation trends in the ‘Marathon Swim’ in Lake Zurich are presented in Table 1. Between 2002 and 2012, a total of 300 swimmers, including 111 women (37%) and 189 men (63%), finished the race. Twelve women (10.8%) and 68 men (36.0%) started in the wetsuit category, 99 women (89.2%) and 121 men (64.0%) started without wearing a wetsuit. For women, no change in participation across years was recorded (Figure 1a), whereas for men, the number in finishers competing with wetsuit increased linearly (Figure 1b). Overall, the number of finishers competing with and without a wetsuit increased linearly (Figure 1c).

**Table 1:** Number of participants in the ‘Marathon Swim’ in Lake Zurich (26.4 km) and in the ‘LOST-Race’ (3.8 km)

**Distance**	**Total**	**Total with wetsuit**	**Total without wetsuit**	**Total men**	**Men with wetsuit**	**Men without wetsuit**	**Total women**	**Women with wetsuit**	**Women without wetsuit**
**26.4 km**	300	80 (26.7%)	220 (73.3%)	189 (63.0%)	68 (36.0%)	121 (64.0%)	111 (37.0%)	12 (10.8%)	99 (89.2%)
**3.8 km**	284	202 (71.1%)	82 (28.9%)	173 (60.9%)	122 (70.5%)	51 (29.5%)	111 (39.1%)	80 (72.1%)	31 (27.9%)

**Figure 2:**
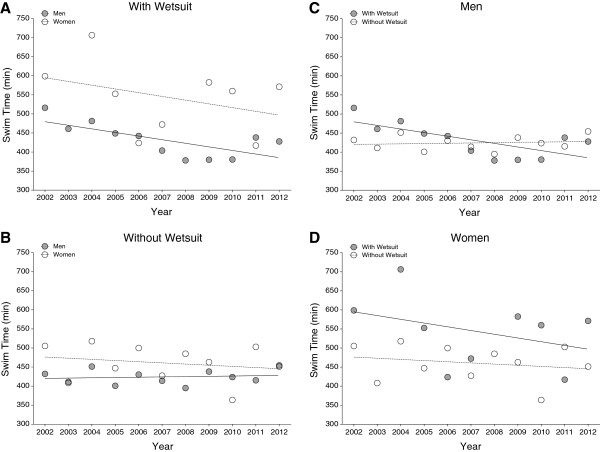
Race times of the annual fastest men and women competing with (Panel A) and without (Panel B) wetsuit ‘Marathon Swim’ in Lake Zurich and the comparison between race times with and without wetsuit for men (Panel C) and women (Panel D).

**Figure 3:**
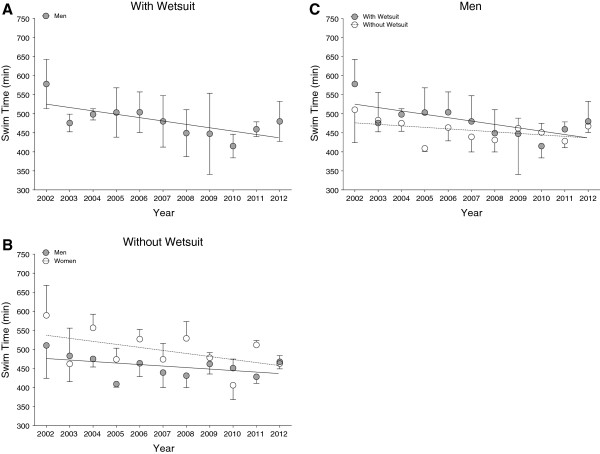
Race times of the annual top three men and women competing with (Panel A) and without (Panel B) wetsuit ‘Marathon Swim’ in Lake Zurich and the comparison between race times with and without wetsuit for men (Panel C).

Figure 2 presents the race times of the annual fastest and Figure 3 of the annual three fastest finishers in the ‘Marathon Swim’ in Lake Zurich. Race times of the annual fastest and the annual three fastest men wearing a wetsuit and race times of the annual three fastest women without wetsuits decreased significantly across years (Table 2). There was no decrease in race times for the annual fastest men competing without a wetsuit and for the annual fastest women with and without a wetsuit over the last decade (Table 2).

**Table 2:** Multi-level regression analyses for change in performance across years for men and women (Model 1) with correction for multiple finishes (Model 2) and with correction for multiple finishes and age of the participants (Model 3)

**Model**	***β***	**SE (*β*)**	**Stand. ß**	**T**	***P***
**Annual fastest men competing with wetsuit**					
**1**	−9.425	3.189	−0.702	−2.955	0.016
**2**	−9.425	3.189	−0.702	−2.955	0.016
**3**	−9.058	3.376	−0.674	−2.683	0.028
**Annual fastest men competing without wetsuit**					
**1**	0.764	1.913	0.132	0.399	0.699
**2**	0.764	1.913	0.132	0.399	0.699
**3**	0.983	2.243	0.170	0.438	0.673
**Annual fastest women competing with wetsuit**					
**1**	−9.791	9.556	−0.361	−1.025	0.340
**2**	−9.791	9.556	−0.361	−1.025	0.340
**3**	−0.323	−0.935	0.386	−0.357	0.991
**Annual fastest women competing without wetsuit**					
**1**	−3.065	4.682	−0.213	−0.655	0.529
**2**	−3.065	4.682	−0.213	−0.655	0.529
**3**	0.204	0.670	0.522	0.231	0.710
**Annual fastest three men competing with wetsuit**					
**1**	−8.845	3.109	−0.455	−2.845	0.008
**2**	−8.845	3.109	−0.455	−2.845	0.008
**3**	−9.848	2.910	−0.507	−3.385	0.002
**Annual fastest three men competing without wetsuit**					
**1**	−3.945	2.366	−0.287	−1.668	0.105
**2**	−3.945	2.366	−0.287	−1.668	0.105
**3**	−4.117	2.500	−0.299	−1.647	0.110
**Annual fastest three women competing with wetsuit**					
**1**	−1.762	9.149	−0.061	−0.193	0.851
**2**	−1.762	9.149	−0.061	−0.193	0.851
**3**	−3.298	8.674	−0.114	−0.380	0.713
**Annual fastest three women competing without wetsuit**					
**1**	−7.956	2.994	−0.431	−2.657	0.012
**2**	−7.956	2.994	−0.431	−2.657	0.012
**3**	−6.875	3.035	−0.372	−2.265	0.031

**Figure 4:**
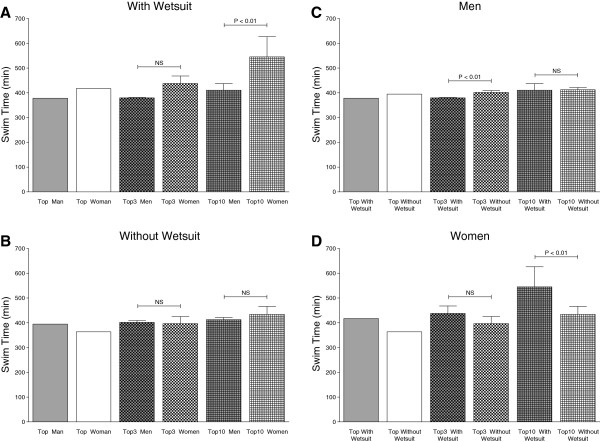
Race times of the top ever, top three ever and top ten ever men and women in the ‘Marathon Swim’ in Lake Zurich competing with (Panel A) and without (Panel B) wetsuit as well as the comparison between men (Panel C) and women (Panel D) competing with and without wetsuit.

Wearing a wetsuit had no effect on overall race time regarding the gender. Men in the wetsuit category finished the race within 534 ± 82 min while men competing without wetsuit had an average race time of 551 ± 89 min (p > 0.05, 3.1%). Women in the wetsuit category swam the 26.4 km in 572 ± 94 min while women competing without a wetsuit achieved an average race time of 589 ± 105 min (p > 0.05, 2.8%). However, race times for men wearing wetsuits were faster than race times for women wearing wetsuits. This finding was non-significant for the top three swimmers (379.4 ± 1.3 min *versus* 396.4 ± 28.8 min, 4.5%, p > 0.05) but significant for the top ten swimmers (410.6 ± 26.7 min *versus* 544.9 ± 81.3 min, 32.7%, p < 0.01) wearing wetsuits (Figure 4a). Male top three (401.6 ± 7.2 min *versus* 396.4 ± 28.8 min, 1.2%, p > 0.05) and male top ten (412.6 ± 9.02 min *versus* 434.2 ± 31.8 min, 5.2%, p > 0.05) swimmers competing without wearing wetsuits were not faster than female swimmers without wearing wetsuits (Figure 4b). Race times of the top three men in the wetsuit category were significantly (p < 0.01) faster (5.9%) than race times of the top three men without wetsuits. Race times of male top and male top three swimmers and race times of top ten swimmers in the wetsuit category and without wetsuits were similar (Figure 4c). The fastest (15%), the top three (10.3%, non-significant) and the top ten (25.5%, significant) race times of all women wearing wetsuits were slower than those of women competing without wetsuits (Figure 4d).

### The LOST-Race

**Figure 5:**
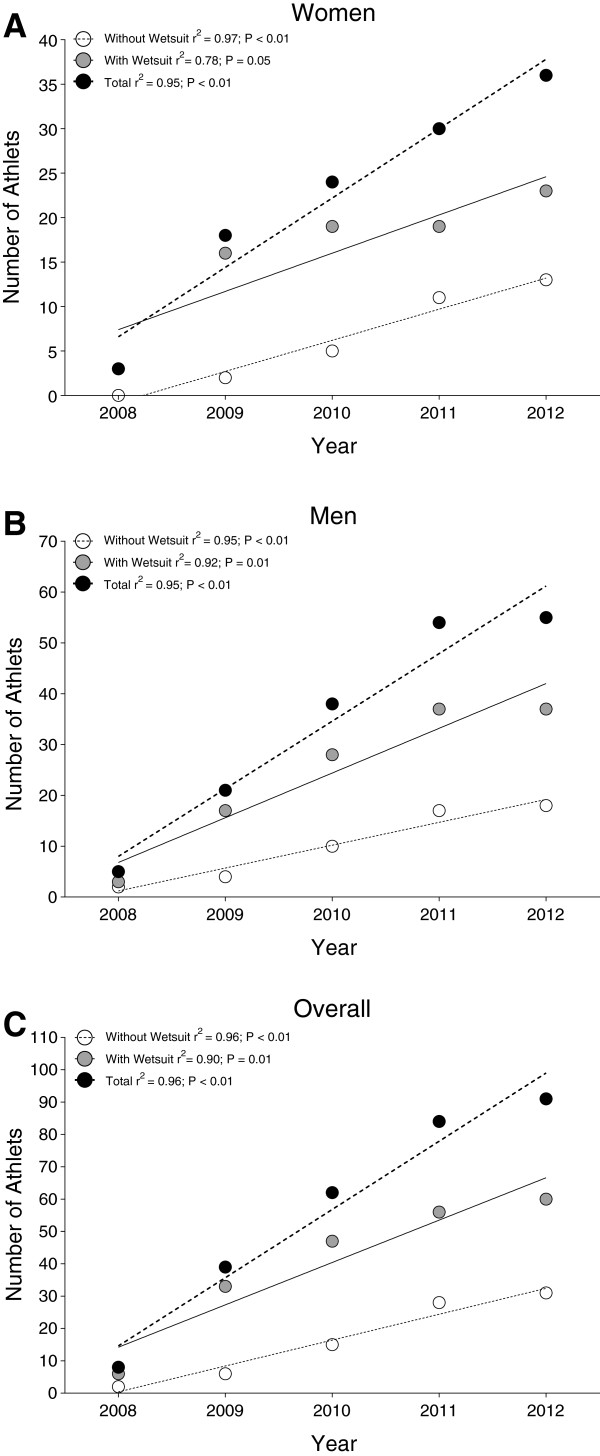
Annual numbers of finishers in the ‘LOST-Race’ competing with and without wetsuit and for women (Panel A), men (Panel B) and overall finishers (Panel C).

The participation trends in the ‘LOST-Race’ are shown in Table 1. In the years 2008 to 2012, 284 swimmers including 111 women (39.1%) and 173 men (60.9%) finished the race. Eighty women (72.1%) and 122 men (70.5%) started in the wetsuit category, 31 women (27.9%) and 51 men (29.5%) started without wearing a wetsuit. The number of finishers in the ‘LOST-Race’ increased significantly since the first event in the year 2008. The increase was consistent for female swimmers in the wetsuit category and without wetsuits (Figure 5a), male swimmers in the wetsuit category and without wetsuits (Figure 5b) and for the overall number of finishers (Figure 5c).

**Figure 6:**
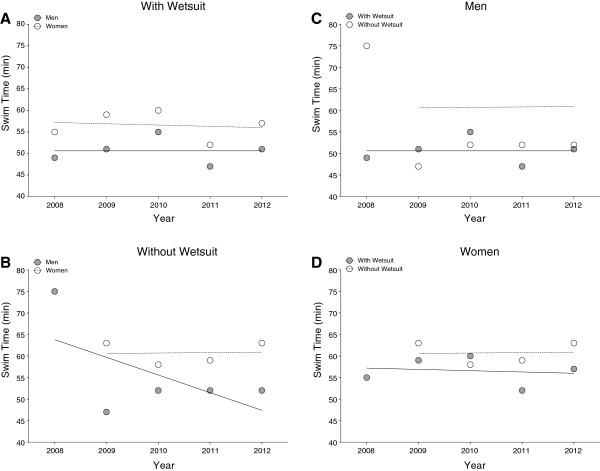
Race times of the fastest men and women competing with (Panel A) and without (Panel B) wetsuit in the ‘LOST-Race’ and the comparison of times for athletes competing with and without wetsuit for men (Panel C) and women (Panel D).

**Figure 7:**
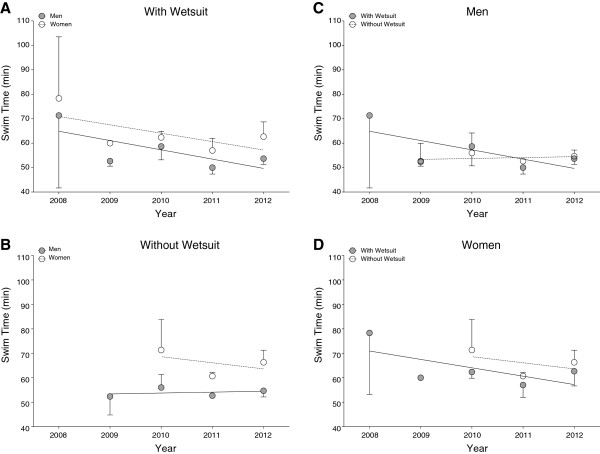
Race times of the top three men and women competing with (Panel A) and without (Panel B) wetsuit in the ‘LOST-Race’ and the comparison of times for athletes competing with and without wetsuit for men (Panel C) and women (Panel D).

Figure 6 presents the race times of the annual fastest and Figure 7 of the annual three fastest finishers in the ‘LOST-Race’. During the investigated years, race times of the annual top three male swimmers without wetsuits decreased significantly while race times of all other groups remained stable (Table 3).

**Table 3:** Multi-level regression analyses for change in performance across years for men and women (Model 1) and with correction for multiple finishes (Model 2)

**Model**	***β***	**SE (*β*)**	**Stand. ß**	**T**	***P***
**Annual fastest men competing with wetsuit**					
**1**	0.000	1.083	0.000	0.000	1.000
**2**	0.000	1.083	0.000	0.000	1.000
**Annual fastest men competing without wetsuit**					
**1**	−4.100	3.272	−0.586	−1.253	0.299
**2**	−4.100	3.272	−0.586	−1.253	0.299
**Annual fastest women competing with wetsuit**					
**1**	−0.300	1.159	−0.148	−0.259	0.813
**2**	−0.300	1.159	−0.148	−0.259	0.813
**Annual fastest women competing without wetsuit**					
**1**	0.100	1.439	0.049	0.070	0.951
**2**	0.100	1.439	0.049	0.070	0.951
**Annual fastest three men competing with wetsuit**					
**1**	−3.800	2.419	−0.399	−1.571	0.140
**2**	−3.800	2.419	−0.399	−1.571	0.140
**Annual fastest three men competing without wetsuit**					
**1**	−4.717	2.040	-.555	−2.313	0.039
**2**	−4.717	2.040	-.555	−2.313	0.039
**Annual fastest three women competing with wetsuit**					
**1**	−3.433	2.193	-.398	−1.566	0.141
**2**	−3.433	2.193	-.398	−1.566	0.141
**Annual fastest three women competing without wetsuit**					
**1**	−0.775	2.191	−0.117	−0.354	0.732
**2**	−0.775	2.191	−0.117	−0.354	0.732

**Figure 8:**
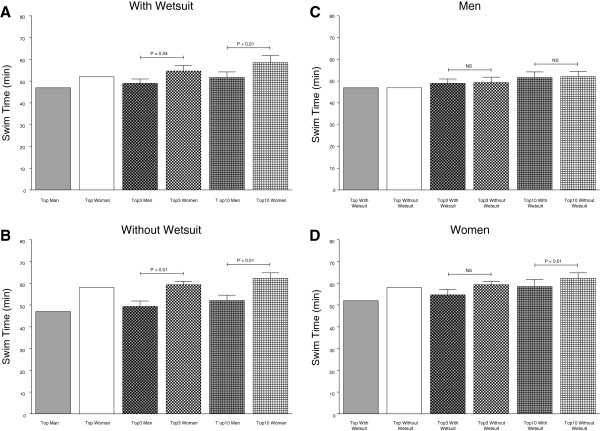
Race times of the top ever, top three ever and top ten ever men and women in the ‘LOST-Race’ competing with (Panel A) and without (Panel B) wetsuit and the comparison between men (Panel C) and women (Panel D) competing with and without wetsuit.

Men in the wetsuit category finished the race within 73 ± 16 min. Men competing without wetsuits achieved an average race time of 73 ± 19 min (p > 0.05, 0.0%). Women in the wetsuit category finished within 79 ± 16 min while women competing without wetsuits finished within 80 ± 19 min (p > 0.05, 1.2%). Men in the wetsuit category swam the 3.8 km generally faster than women. This finding was consistent for the fastest swimmers (47.0 min *versus* 58.0 min) as well as for the top three swimmers (49.0 ± 2.0 min *versus* 54.7 ± 2.6 min, 11.6%, p = 0.04) and the top ten swimmers (51.7 ± 2.5 min *versus* 58.5 ± 3.2 min, 13.2%, p < 0.01) in the wetsuit category (Figure 8a). Also the male top (47.0 ± 0 min *versus* 58.0 ± 0 min), male top three (49.3 ± 2.5 min *versus* 59.3 ± 1.5 min, 20.3%, p < 0.01) and male top ten swimmers (52.1 ± 2.4 min *versus* 62.3 ± 2.5 min, 19.6%, p < 0.01) competing without wetsuit were faster than the female top swimmers (Figure 8b). When comparing race times of the fastest, the top three and the top ten male swimmers, equal race times for swimmers in the wetsuit category and swimmers competing without wetsuits were observed (Figure 8c). In contrast, the fastest female swimmers (52.0 ± 0 min *versus* 58.0 ± 0 min) as well as the top three (54.7 ± 2.5 min *versus* 59.3 ± 1.5 min, 8.5%, p > 0.05) and the top ten female swimmers (58.5 ± 3.2 min *versus* 62.3 ± 25 min, 6.5%, p < 0.01) competing in the wetsuit category were faster than swimmers without wetsuits (Figure 8d).

## Discussion

The aim of the study was to compare for both male and female open-water swimmers the performance while competing with and without wearing a wetsuit in a long-distance (*e.g.* 3.8 km) and in an ultra-long-distance swimming (*e.g.* 26.4 km) event.

### Swimmers wearing wetsuits were not faster than swimmers without wetsuits

The most important finding was that open-water swimmers competing while wearing a wetsuit were not generally faster in both the long and the ultra-long-distance swimming event than swimmers without wearing wetsuits depending upon whether the fastest, the three fastest or the ten fastest swimmers were considered. This finding contradicts existing literature [21,23] reporting better performances for swimmers competing while wearing wetsuits in short to middle distances between a standardized 500 m test track and 1,500 m.

In the ‘Marathon Swim’ in Lake Zurich, only the overall top three men wearing a wetsuit were swimming faster than swimmers without a wetsuit. Considering the overall fastest and the top ten as well as the annual fastest, the top three and the top ten male swimmers, race times were similar. Also in the ‘LOST-Race’, male swimmers wearing wetsuits were competing at the same speed like swimmers without a wetsuit. Female swimmers wearing a wetsuit performed significantly faster in 3.8 km while women wearing wetsuits swam significantly slower in 26.4 km than women without a wetsuit.

Our hypothesis that swimmers competing in a wetsuit would be faster in long and ultra-long-swimming distances was based on existing studies suggesting a positive effect of wearing a wetsuit on swimming performance [22,23,25,26]. All those studies were conducted in indoor pools with standardized water temperatures either covering short to middle distances on a standardized 500 m test track up to 1,500 m or standardized swimming times. It has been shown by Cordain and Kopriva [23] that wetsuits lift the body in a more horizontal position by providing higher buoyancy while swimming in indoor-pools. Thus less power is required for swimmers to maintain the swimming position leaving more energy to be expended for propulsion [23].

In Lake Zurich and Lake Ontario water temperatures were constantly over 20°C when the ‘Marathon swim’ [13] and the ‘LOST-Race’ [19] were held. Cordain and Kopriva [23] reported that swimmers competing in a pool with rather warm temperatures of ~26°-28°C complained about feeling too hot while swimming in a wetsuit. This problem was observed in a 1,500 m distance more often than in a 400 m distance [23]. Lowdon *et al.*[35] suggested a temperature of 17.7°C as for a positive effect of wetsuits on swimming race times because of their insulating features. Race times of the annual top three male swimmers without wetsuits on the 3.8 km long distance decreased significantly over the last years while race times of male top three swimmers wearing wetsuits remained unchanged. Thus, we assume that swimmers wearing wetsuits could have been disturbed by the reduced ability of the body to dissipate heat on that long distance when wearing wetsuits and therefore been unable to show an optimal performance.

In contrast, the race times of the annual top and top three swimmers on the ultra-long track decreased during the last decade and the top three male swimmers wearing wetsuits competing in the 26.4 km swam significantly faster than swimmers without wearing a wetsuit. Tipton *et al.* showed a decrease of body core temperature of 0.75°C per hour for swimmers performing in water with a temperature of 18°C without wetsuit [36]. We assume that even when wearing wetsuits while swimming in water of ~20°C for ~6-7 hours there will be a decrease of body core temperature leading to a better comfort and thus enabling to perform better.

### Different clothing in ultra-endurance and Ironman-triathletes

The ‘Marathon Swim’ in Lake Zurich was initially introduced as a training event. Ultra-endurance swimmers still use it for preparation for more popular events like the ‘English Channel Swim’ [37]. Additionally, a lot of participants in the event are Ironman triathletes who were preparing for the swimming split of their competitions such as the ‘Ironman Switzerland’ and the ‘Ironman 70.3’ Rapperswil-Jona taking place in the Lake Zurich [38].

The use of wetsuits is not allowed in ultra-distance swimming events like the ‘English Channel Swim’. In contrast, wearing a wetsuit is permitted in the most important Ironman competitions including the ’Ironman 70.3 Series’ and the ‘Ironman World Championship’ as long as water temperature is 24.5°C or colder. Therefore, we suppose that most participants wearing a wetsuit when swimming in the ‘Marathon Swim’ in Lake Zurich were Ironman triathletes while ultra-endurance swimmers will rather swim without wetsuit when competing over the 26.4 km.

Knechtle *et al.*[37] compared the anthropometry of ultra-swimmers and Ironman athletes and showed that male and female ultra-swimmers have a higher body mass, a higher body mass index and a higher percentage of body fat than Ironman triathletes leading to a higher buoyancy and a better insulation against cold water temperatures. Leaner athletes thus were supposed to benefit more from wearing wetsuits than fatter swimmers [23]. On the other hand Knechtle *et al.*[37] suggested that other factors like training have a larger influence on swimming performance than anthropometric features. Triathletes have to prepare for three disciplines and as the swimming split in an Ironman triathlon is only 3.8 km and even in a Triple-Iron ultra-triathlon only 11.4 km, ultra-swimmers may be better trained for a swimming event covering 26.4 km [32]. It has been reported that female and male long-distance open-water swimmers competing in ‘Marathon Swim’ in Lake Zurich swam 16.2 ± 8.5 and 15.6 ± 13.3 km weekly, respectively, at a mean speed of 3.4 ± 0.6 and 3.5 ± 0.5 km/h, respectively [32]. In contrast, female and male Ironman triathletes competing in ‘Ironman Switzerland’ swam 5.5 ± 2.4 and 6.7 ± 3.0 km weekly, respectively, at a mean speed of 2.1 ± 0.8 and 2.7 ± 0.6 km/h, respectively [39]. Most probably, also swimmers competing in a 3.8 km swim will train less that ultra-swimmers competing in a 26.4 km ultra-swim. The combination of better anthropometric features and better specialized training of ultra-swimmers for the event could have been the reason why the advantages of wearing a wetsuit have been compensated in this event.

### Female athletes seemed to benefit more from wearing wetsuits than male swimmers

In the ‘LOST-Race’, female swimmers of all investigated performance levels were faster when wearing wetsuits than swimmers competing without wetsuits. In contrast, the race times of male swimmers were similar when competing with and without wetsuits. Swimming without wetsuits on the 3.8 km track resulted in a difference in race time of 19.6% in favour for male swimmers. In contrast, when wearing wetsuits, the top ten male swimmers performed only 11.9% faster than the top ten female athletes. This data is coinciding with the study of Lepers reporting a sex difference of 12% in the swimming split in an Ironman competition such as ‘Ironman Hawaii’ [40].

An explanation for the higher benefit of female swimmers from the use of wearing a wetsuit could be the anthropometric differences between the sexes. Male swimmers have a higher stroke volume and therefore a higher cardiac output than female athletes [41]. The vascularisation of the skeletal muscles is better in men because of the higher blood volume [41]. These factors result in a higher oxygen capacity for male swimmers. Furthermore, the maximum oxygen uptake is ~15-25% higher in men [41]. These factors in combination with higher androgen levels and a higher skeletal muscle mass lead to a higher strength and a higher endurance of male compared to female swimmers [10]. Wetsuits lift the body in a more horizontal position by providing higher buoyancy. Therefore, less power is required to maintain the swimming-position leaving more energy to be expended in propulsion [23]. Because of the discussed physiological and anthropometrical features, female swimmers will be able to save more energy by using the floating features of wetsuits. Therefore, female swimmers might improve race times more than male swimmers. It is known that wetsuits lift the body in a more horizontal position by providing higher buoyancy. However, when we analyse the compression of the body due to the suit, one can expect that buoyancy could decrease as body mass is the same but body volume becomes lower. Regarding this aspect, buoyancy may decrease in both women and men. The reduction might be relatively higher in men with lower body fat then in women.

Female winners of the ‘Marathon Swim’ in Lake Zurich covering 26.4 km and wearing wetsuits were ~20.3% slower than male winners wearing wetsuits, while there was only a difference of ~8.0% between female and male winners without wearing a wetsuit. This finding contradicts our hypothesis that female swimmers would benefit more from wearing a wetsuit than male athletes. In the period from 2002 to 2012, a total number of only 12 female swimmers wearing a wetsuit finished the race leading to a mean of one annual female finisher wearing a wetsuit. Therefore, the statistical analyses concerning the sex difference in finishers wearing a wetsuit are most probably biased by the small number of female finishers. Future studies need to compare large groups of athletes to confirm the present findings.

Female swimmers have a lower skeletal muscle mass and a higher fat mass than male swimmers. This might lead to the assumption that female swimmers would present a more stable horizontal position in the water due to better buoyancy capability. On the other side, men have lower body fat and a higher skeletal muscle mass, so wetsuits might be more beneficial for male swimmers. Indeed, Cordain and Kopriva [23] suggested that the increase in performance by the increase in buoyancy might be of more benefit for leaner than for fatter subjects. This consideration might also be supported by the very recent finding that female open-water ultra-swimmers competing in the 46 km ‘Manhattan Island Marathon Swim’ with water temperatures <20°C were faster than male swimmers [17]. The increase in buoyancy might me more important than the reduction in drag. Toussaint *et al.*[24] showed that the effect of wetsuit on drag was not different between lighter female swimmer and heavier male swimmers. One might argue that men wearing a wetsuit might be faster than women in ~50 km at water temperatures <20°C.

### Limitations of the study and implications for future research

This study was designed as a retrospective analysis of the performances in the ‘Marathon Swim’ in Lake Zurich and the ‘LOST-Race’. We compared race times of swimmers wearing wetsuits with race times of swimmers competing without wearing wetsuits. This comparison could be biased by various factors like missing physiologic features [40], missing experience in ultra-endurance swimming and missing training volume [37]. A part of the participants in the ‘Marathon Swim’ in Lake Zurich are triathletes specialized in shorter swimming distances and therefore probably slower in this ultra-long distance. From the obtainable data we have no further information about the composition of the analysed groups. The resulting bias is a limitation of this study. Furthermore, the small number of female finishers in the ‘Marathon Swim’ in Lake Zurich might be a possible bias for the statistical analyses. Future studies would need to compare swimming performance in open-water ultra-distance swimming of distances of ~50 km at water temperatures <20°C. Men with lower body fat might benefit from wearing a wetsuit under these conditions.

## Conclusion

The present results suggest that swimmers competing in long and ultra-long-distance open-water swimming events could benefit from wearing wetsuits depending upon whether the fastest, the three fastest or the ten fastest swimmers were considered. This is consistent with existing literature reporting better performances in swimmers wearing wetsuits while swimming in indoor pools on short to middle distances. However, the present data suggest that the benefit of wearing wetsuits while competing in open-water swimming events may depend on various factors like water temperature and race distance as well as anthropometric and physiological features. Additionally, it depends upon the size of the analysed sample (*i.e.* the fastest, the three fastest or the ten fastest swimmers). The benefit of wearing wetsuits seemed also to be different in male and female swimmers. In order to obtain reliable information about the effect of wearing a wetsuit on swimming performance in long-distance and ultra-long-distance open-water swimming, additional studies should be conducted. It would be interesting to compare swimming times of the same male and female open-water ultra-distance swimmers once wearing wetsuits and once performing without wearing wetsuits. These studies should be conducted on different distances and with different water temperatures. As an additional procedure measurement of body temperature during the swim should be included. When the race conditions are known the obtained data could facilitate ultra-endurance swimmers and ironman swimmers the decision if wearing a wetsuit in a competition would be beneficial.

## Competing interests

The authors declare that they have no competing interests.

## Authors’ contributions

SU collected the data and drafted the manuscript; CR performed the statistical analyses; TR and RL participated in the design of the study and helped drafting the manuscript; and BK helped in interpretation of the results and drafting the manuscript. All authors read and approved the final manuscript.

## Pre-publication history

The pre-publication history for this paper can be accessed here:

http://www.biomedcentral.com/2052-1847/6/20/prepub
